# New Perspectives in the Renin-Angiotensin-Aldosterone System (RAAS) IV: Circulating ACE2 as a Biomarker of Systolic Dysfunction in Human Hypertension and Heart Failure

**DOI:** 10.1371/journal.pone.0087845

**Published:** 2014-04-01

**Authors:** Katalin Úri, Miklós Fagyas, Ivetta Mányiné Siket, Attila Kertész, Zoltán Csanádi, Gábor Sándorfi, Marcell Clemens, Roland Fedor, Zoltán Papp, István Édes, Attila Tóth, Erzsébet Lizanecz

**Affiliations:** 1 Division of Clinical Physiology, Institute of Cardiology, Medical and Health Science Centre, University of Debrecen, Debrecen, Hungary; 2 Institute of Cardiology, Medical and Health Science Centre, University of Debrecen, Debrecen, Hungary; 3 Institute of Surgery, Medical and Health Science Centre, University of Debrecen, Debrecen, Hungary; 4 Research Centre for Molecular Medicine, Medical and Health Science Centre, University of Debrecen, Debrecen, Hungary; School of Pharmacy, Texas Tech University HSC, United States of America

## Abstract

**Background:**

Growing evidence exists for soluble Angiotensin Converting Enzyme-2 (sACE2) as a biomarker in definitive heart failure (HF), but there is little information about changes in sACE2 activity in hypertension with imminent heart failure and in reverse remodeling.

**Methods, Findings:**

Patients with systolic HF (NYHAII-IV, enrolled for cardiac resynchronisation therapy, CRT, n = 100) were compared to hypertensive patients (n = 239) and to a healthy cohort (n = 45) with preserved ejection fraction (EF>50%) in a single center prospective clinical study. The status of the heart failure patients were checked before and after CRT. Biochemical (ACE and sACE2 activity, ACE concentration) and echocardiographic parameters (EF, left ventricular end-diastolic (EDD) and end-systolic diameter (ESD) and dP/dt) were measured.

sACE2 activity negatively correlated with EF and positively with ESD and EDD in all patient's populations, while it was independent in the healthy cohort. sACE2 activity was already increased in the hypertensive group, where signs for imminent heart failure (slightly decreased EF and barely increased NT-proBNP levels) were detected. sACE2 activities further increased in patients with definitive heart failure (EF<50%), while sACE2 activities decreased with the improvement of the heart failure after CRT (reverse remodeling). Serum angiotensin converting enzyme (ACE) concentrations were lower in the diseased populations, but did not show a strong correlation with the echocardiographic parameters.

**Conclusions:**

Soluble ACE2 activity appears to be biomarker in heart failure, and in hypertension, where heart failure may be imminent. Our data suggest that sACE2 is involved in the pathomechanism of hypertension and HF.

## Introduction

The renin–angiotensin system (RAS) is a central regulator of cardiovascular and renal functions and plays an important role in the pathophysiology of heart failure [1] [2]. Soluble Angiotensin converting enzyme 2 (sACE2) is a recently discovered homologue of ACE. It is a monocarboxypeptidase generating Ang-(1–9) from Ang-I [3] and Ang-(1–7) from Ang-II. Ang-(1–7) is a biologically active metabolite of the RAS acting through the G-protein-coupled Mas receptor [4]. Ang-(1–7) is capable of reducing myocardial oxidative stress and pathological remodeling [5]. Mas receptor can hetero-oligomerize with AT1R acting as a physiological antagonist of AngII [6].

In the heart, sACE2 is expressed in various cell types including fibroblasts, cardiomyocytes and endothelial cells [7]. Although sACE2 is a plasma membrane-bound ectoenzyme, a soluble active form of the protein was also found in plasma and urine [8]. Tumor necrosis factor alpha converting enzyme (TACE/ADAM17) is the sheddase responsible for the ectodomain cleavage and shedding of sACE2 [9].

Opposite to the AngI-ACE-AngII-AT1R pathway sACE2 may provide a vasoprotective/antiproliferative mechanism resulting in the counter-regulation of the RAS [10]. In accordance, previous animal data have shown that transgenic sACE2 overexpression attenuates hypertension [11] [12]. Suppression of sACE2 expression again established it as a negative regulator of the RAS in blood pressure control [11] [13] [14]. Moreover, sACE2 polymorphisms were related to hypertension in different human populations [15] [16] [17]. Nonetheless, the expression and activity of sACE2 in human hypertension has not been addressed directly yet.

In contrast to hypertension, sACE2 has already been studied in animal and human HF suggesting a protective role for this enzyme [18]. Targeted disruption of sACE2 in mice results in severe cardiac contractility defect, increased plasma and heart AngII levels leading to cardiac dysfunction. Absence of sACE2 causes stress activation of the myocardial NADPH oxidase system and leads to severe adverse myocardial remodeling and dysfunction [19]. It was suggested that myocardial sACE2 gene expression is increased in patients with left ventricular dysfunction [20] and TACE is also upregulated in HF [9]. Loss of sACE2 worsened the pathological remodeling and resulted in a rapid progression to systolic dysfunction and HF [21].

Epelman et al. showed that increased sACE2 activity is associated with more advanced HF and that elevated sACE2 activity could predict adverse cardiac events [8]. Lehmann et al. recently observed higher sACE2 activity in HF-patients experiencing ventricular arrhythmias and appropriate defibrillator-intervention [22]. Whether these considerable correlations make sACE2 activity suitable as a novel biomarker of heart failure is still not settled.

Growing evidence exists for irrefutable importance of sACE2 in the pathophysiology of HF, however there is little information about changes in sACE2 activity during the progression of the disease as well as about reverse changes under medical therapy such as Cardiac Resynchronization Therapy (CRT).

Here we report a single center, prospective clinical study to establish a relationship between circulating ACE, sACE2 and clinical parameters, such as hypertension or cardiac performance. Considering that use of terms related to ACE enzyme-activity and enzyme-level occurs inconsistently in the literature, we performed parallel ACE enzyme activity and enzyme concentration measurements. We have studied these relationships in patients with severe remodeling and during reverse remodeling when improved systolic function was achieved by biventricular pacemaker device therapy. sACE2 activity was measured in hypertensive patients for the first time, and sACE2 was identified as a biomarker of imminent heart failure, when cardiac ejection fraction is above 50%, but deterioration of cardiac performance is expected (e.g. in patients with hypertension).

## Methods

### Study populations

Written informed consent has been obtained from the patients and all clinical investigation has been conducted according to the principles expressed in the Declaration of Helsinki. The study has been approved by the Regional and Institutional Ethics Committee, Medical and Health Science Center, University of Debrecen, (UDMHSC REC/IEC number: 3261–2010) and by the Medical Research Council of Hungary.

A single center, prospective clinical study was performed at the Institute of Cardiology of the University of Debrecen to investigate the relationship between serum sACE2 activity and cardiovascular pathologies, among other parameters.

There were three study groups. Healthy individuals (n = 45) without any cardiovascular pathology or medication were recruited with normal cardiac morphology and left ventricular ejection fraction above 50%.

A second group of 239 hypertensive patients (systolic blood pressure above 140 mmHg and/or diastolic blood pressure above 90 mmHg at the time of the diagnosis of the disease) was established. This group was characterized by preserved ejection fraction (above 50%) besides optimal antihypertensive therapy according to the national guidelines (Table 1).

**Table 1:** Clinical characteristics of the study patients.

Variables	Healthy people n = 45	Hypertensive patients n = 239	Heart failure (HF-CRT before) n = 100
Age, years (mean±SD)	30.2±8.7	62.3±9.6*	63.5±10.8* #
Gender: men, %	45	59	79
Echocardiographic mesurements (mean±SD)			
EF, %	62.0±4.2	56.6±4.5*	28.3±5.4* #
EDD, mm	48.6±3.9	51.7±5.7*	67.2±9.2* #
ESD, mm	29.9±3.6	33.4±5.4*	56.3±9.3* #
dP/dt, mmHg/s	-	-	498.2±27.1
Cardiovascular risk factors, %			
Hypertension	0	100	69
Diabetes	0	23	26
Dyslipidemia	0	70	71
Medication at enrollment, %			
ACE inhibitor	0	83	89
ARB	0	15	11
β-Blocker	0	79	100
Aldosterone antagonist	0	6	89
Diuretics	0	53	91
NT-proBNP level, pmol/L	6.5±4.8	32.5±69.4*	394.6±522.7* #

*:healthy vs. others.

# :hypertensive vs. CRT before.

A third group of 100 patients with severe left ventricular systolic dysfunction with indication of cardiac resynchronization therapy (heart failure, HF) were also enrolled into the study (HF - CRT before). Patients were selected for CRT according to the current ESC guideline related to pharmaceutical and device therapy of systolic heart failure [23], [24] Till the date 65 patients fulfilled the first visit between 6 and 9 months (HF - CRT after), so statistical analyses and diagrams contain exclusively their data. Significant mitral regurgitation was present in 22 patients, enabling dP/dt measurements.

Examinations were performed at the enrollment (healthy group), at regular visits (hypertensive group) or just before (HF - CRT before group) and between 6 and 9 months after CRT device implantation (HF – CRT after group). Each visit included physical examination with assessment of New York Heart Association (NYHA) functional stage, echocardiographic measurements and blood sample collection for biochemical measurements. Cardiovascular risk assessment comprised age, sex, hypertension, hypercholesterinaemia, diabetes mellitus or ischaemic cardiomyopathy (Table 1). NYHA classification was performed by independent clinicians who were not aware of echocardiographic data. Medical reports and medication history were obtained from all patients.

### Echocardiographic measurements

Transthoracic echocardiography was performed using an Accuson Sequoia (Siemens AG, Germany) echocardiograph. Two dimensional and Doppler imaging was performed in standard parasternal and apical views. The left ventricular ejection fraction (EF) was measured by M mode left ventricular dimensional method. Preserved EF cutoff was >50%. Values for dP/dt (a functional parameter which is related to left ventricular contractility) were determined in patients with severe mitral regurgitation. Two experienced cardiologists unaware of the biochemical data performed the echocardiographic measurements.

### Blood sample collection

Blood samples were collected by using a standard aseptic technique. Native blood was incubated for 60 minutes at room temperature. Serum fractions were separated by centrifugation (1,500 g, 15 min) and kept in a freezer (−20°C) until the measurements.

### Measurement of serum ACE2 activity

The sACE2 activity measurement was performed using a specific quenched fluorescent substrate as previously described with some modifications [25] [26] [27]. The reaction mixture (200 μL) contained 20 μl serum, 80 μL buffer and 100 μl (50 μM) sACE2-specific fluorescent substrate (7-methoxycoumarin-4-yl)acetyl-Ala-Pro-Lys(2,4-dinitrophenyl)-OH [Mca-APK(Dnp)] (EZ Biolab, Carmel, USA). sACE2 activity was measured by fluorometric assay of the enzymatic cleavage of K(Dnp) from the fluorogenic substrate Mca-APK(Dnp).

The reaction buffer contained a protease inhibitor cocktail 10 μM Bestatin-hidrochloride, 10 μM Z-prolyl-prolinal (Enzo Life Science, Exeter, UK), 5 μM Amastatin-hidrochloride, 10 μM Captopril and 5 mM NaCI, 100 μM ZnCI_2_, 75 mM TRIS HCI, pH 6.5. All chemicals were from Sigma (St. Louis, MO, USA) if not stated otherwise.

The reaction was performed in black 96-well microtiter plates (Greiner Bio-One, Frickenhauser, Germany). The assay was monitored continuously by measuring the increase in fluorescence (excitation wavelength = 320 nm, emission wavelength = 405 nm) upon substrate hydrolysis using a fluorescence microplate reader (NOVOstar; BMG Labtech GmbH, Offenburg, Germany). Initial enzyme activities were determined from the linear rate of fluorescence increase over the 0–120 min time course. The increase in fluorescence was plotted as a function of reaction time and fitted with a linear regression.

sACE2 activity was calculated by the equation:

sACE2 activity = (S/k)*D

where *S* is the rate of observed increase in fluorescence intensity, *k* is the change in fluorescence intensity upon the complete cleavage of 0.1 nmol of Mca-APK(Dnp), and *D* is the dilution of the serum. 1 unit of fluorescence (UF) corresponds to the quantity of enzyme which can degrade 0.1 nmol Mca-APK(Dnp) in 1 hour at 37 °C. The specificity of the sACE2 enzyme activity assay was tested by the specific human sACE2 inhibitor DX600, on a single test sample, where DX600 resulted in a complete inhibition of Mca-APK(Dnp) cleavage (Figure S1). Fits were accepted when r>0.95.

### Measurement of serum angiotensin converting enzyme (ACE) activity

Assessment of ACE activity was based on the spectrophotometric measurement of FAPGG hydrolysis [28] [29]. The reaction mixture (200 μL) contained 50 μL of serum, 0.5 mM FAPGG (N-[3-(2-Furyl)acryloyl]-L-phenylalanyl-glycyl-glycine) (Sigma, St. Louis, MO, USA) substrate, 300 mM sodium chloride, and 25 mM HEPES (4-(2-hydroxyethyl)-1-piperazineethanesulfonic acid) at pH 8.2. Measurement of ACE activity is based on the change in the absorption at 340 nm when FAPGG hydrolyzed to furylacryloyl-L-phenylalaline (FAP) and glycylglycine (GG). The reaction was performed in 96-well plates (Greiner Bio-One, Frickenhauser, Germany). Changes in FAPGG absorbance were detected using a microplate reader (NOVOstar; BMG Labtech GmbH, Offenburg, Germany). Hydrolysis of FAPGG by ACE was recorded in every 5 minutes at 37C°. Optical density values were plotted as a function of reaction time and fitted by linear regression. The fit and the data were accepted when *r*>0.9. ACE activity was calculated by the equation:

ACE activity = −(*S*/*k*)*D,

where *S* is the rate of observed decrease in optical density (1/min), *k* is the change in optical density upon the complete cleavage of 1 nmol of FAPGG, and *D* is the dilution of the serum. One unit (U) of ACE activity represents 1 nmol of FAPGG hydrolysis per minute at 37 °C.

### Measurement of serum ACE concentration

ACE concentration was determined using a Human ACE enzyme-linked immunosorbent assay (ELISA) Development System (catalog No. DY929; R&D System, Inc, Minneapolis, USA) according to the manufacturer's instruction, with minor modifications [30]. In brief, enzyme–linked immunosorbent assay plates were coated with capture antibody diluted to a working concentration of 80 ng/well in Dulbecco's modified phosphate-buffered saline solution (DPBS) (Invitrogen Corp, Carlsbad, CA, USA) overnight at room temperature. The remaining binding sites were blocked with bovine serum albumin (Sigma, St. Louis, MO, USA), 10 mg/mL, dissolved in DPBS. Human serum samples were diluted 100-fold in the same buffer (10 mg/mL of bovine serum albumin in DPBS) and incubated with the immobilized primary antibodies for 2 hours. Capture antibody-bound ACE was labeled using a biotinylated detection antibody, 20 ng/well for 2 hours. Streptavidin-conjugated horseradish-peroxidase (200-fold-diluted stock from the kit) was added to the wells and incubated for 20 minutes. The immunocomplexes were detected with a chromogenic substrate solution containing 0.3 mg/mL TMB (3,3,5′,5′-tetramethylbenzidine), 0.1 μM H_2_O_2_ and 50 mM acetic acid (incubation time was about 20 minutes). Reaction was terminated by addition of 0.5 M HCl and was evaluated by measuring absorbance at 450 nm. ACE concentration was calculated using a calibration curve. The ACE concentration in the samples were measured at least three times to achieve a standard deviation of at most 15%. Serum ACE concentration was given as ng/mL of serum.

### N-Terminal Pro-Brain Natriuretic Peptide (NT-proBNP) measurements

NT-proBNP levels were measured in serum using a commercially available kit (Elecsys proBNP II., Roche Ltd., Mannheim, Germany).

### Statistical analysis

**Figure 1:**
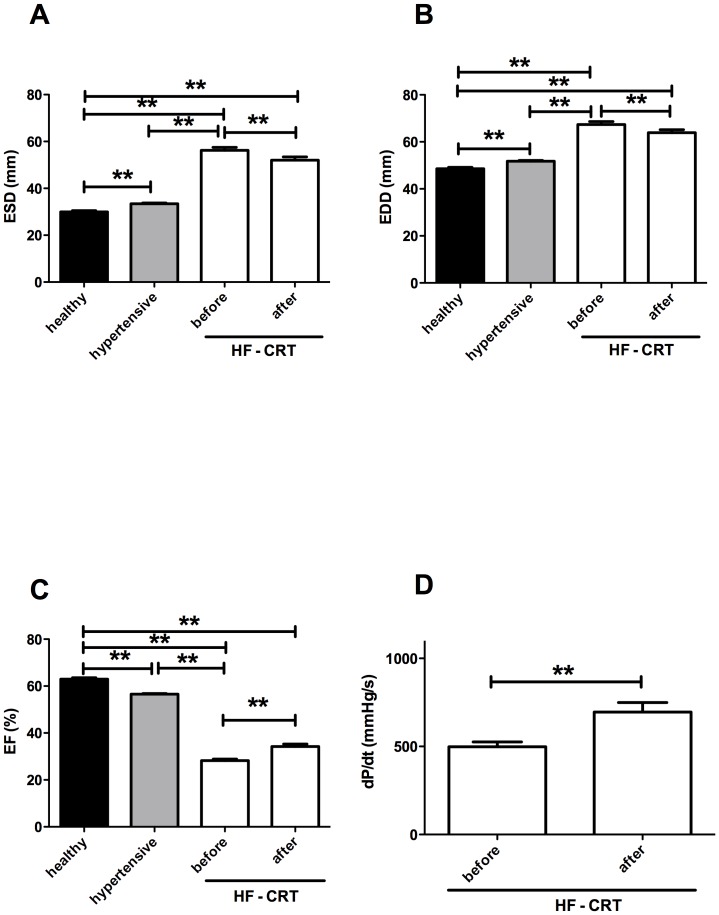
Echocardiographic parameters of the study populations. Left ventricular echocardiographic parameters end systolic diameter (ESD, panel A), end diastolic diameter (EDD, panel B) and ejection fraction (EF, panel C) are shown in healthy people (healthy, n = 45, filled bars), patients with hypertension (hypertensive, n = 239, grey bars) and with severe heart failure (HF - CRT, n = 65, open bars). The kinetics of ventricular contractions (dP/dt) is shown in patients with severe heart failure (panel D, n = 22). Values in patients undergoing cardiac resynchronization therapy are shown before device implantation (HF - CRT, before) and after 6–9 months (HF - CRT, after). Unpaired echocardiographic values were analyzed by the nonparametric Kruskal-Wallis test (two tails, panel A–C). Paired echocardiographic data (CRT before and after, panel A–C and dP/dt, panel D) were evaluated by the nonparametric Wilcoxon matched-pairs signed rank test (two tails). Bars are mean ± S.E.M. Significant differences are labeled by asterisks (** P<0.001).

Unpaired echocardiographic values were analyzed by the nonparametric Kruskal-Wallis test (two tails, Figure 1A–C). With these nonparametric tests there is a difference between the hypertensive and healthy patients groups. Bars are mean ± S.E.M. Significant differences are labeled by asterisks (** P<0.001). Paired echocardiographic data (CRT before and after, Figure 1A–1C) were evaluated by the nonparametric Wilcoxon matched-pairs signed rank test (two tails). The dP/dt parameter was analyzied with paired t-test (two tailes, Figure 1D).

**Figure 2:**
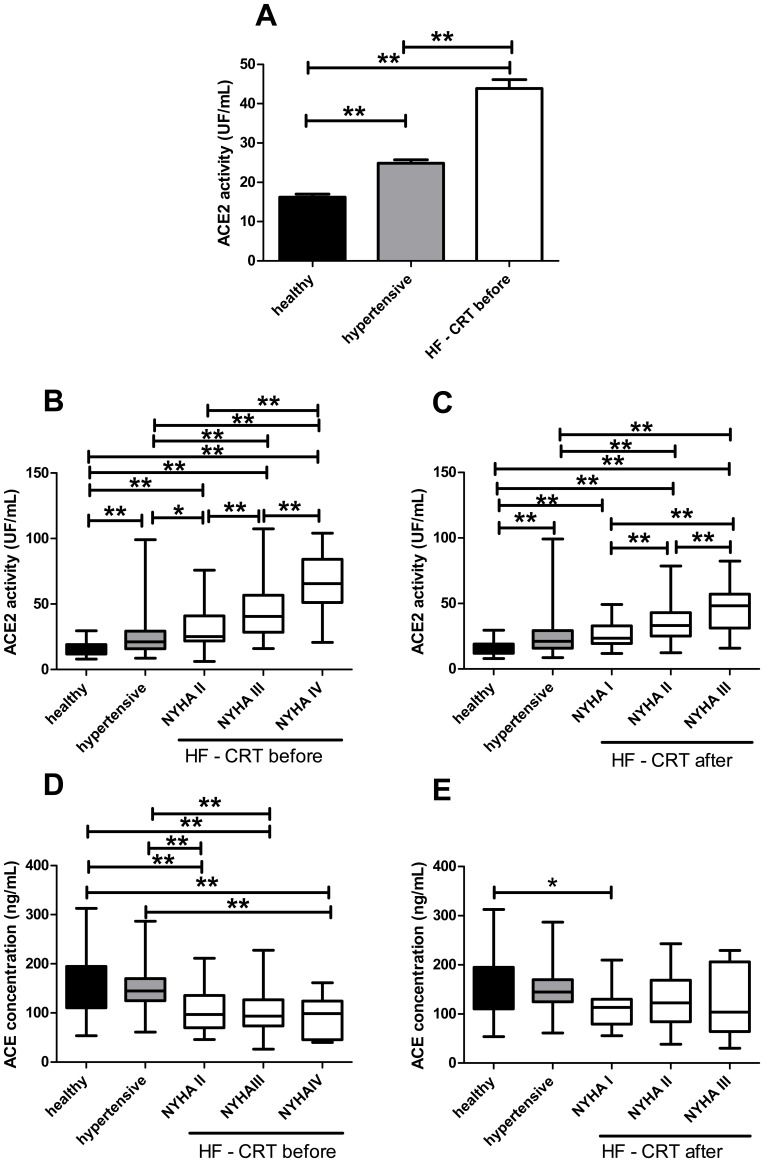
Differences in serum angiotensin converting enzyme activities and concentrations in study populations representing various clinical status of heart failure. Serum ACE2 activity levels were determined in healthy people (healthy, n = 45, filled bar, panel A), patients with no apparent heart failure (hypertensive, n = 239, grey bar, panel A) and in patients with severe systolic heart failure (HF - CRT before, n = 100, open bars, panel A). Patients were also evaluated with respect of their New York Heart Association (NYHA) functional class (HF - CRT before: NYHAII: n = 31; NYHAIII: n = 55; NYHAIV: n = 14, panel B; HF - CRT after: NYHAI: n = 17; NYHAII: n = 37; NYHAIV: n = 11, panel C). Serum ACE concentration was also determined with respect of the New York Heart Association (NYHA) functional class of the patients before (HF – CRT before, panel D) and after CRT (HF – CRT after, panel E). Statistical analyses of healthy people, hypertensive, HF - CRT before and HF – CRT after groups were performed by one-way analysis of variance (ANOVA) followed by Newman-Keuls test for multiple comparisons between groups. Bars are mean ± S.E.M. Significant differences are labeled by asterisks (** P<0.001).

Statistical differences in the biochemical parameters (figures where soluble ACE2 activities are evaluated, Figure 2) were addressed by the parametric ANOVA tests (comparison of all group-pairs, Newman-Keuls post test).

**Figure 3:**
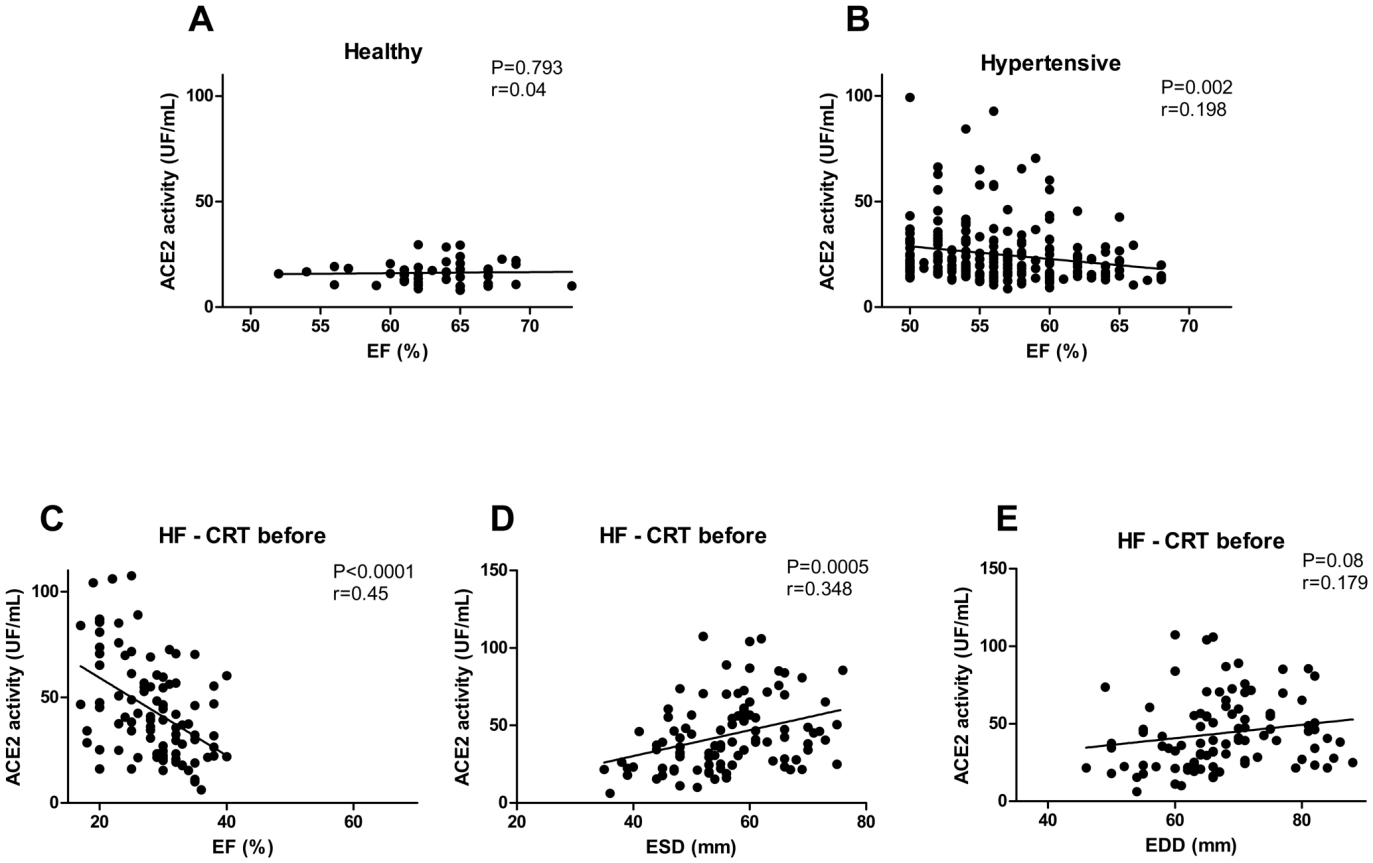
Serum ACE2 activity correlates with left ventricular ejection fraction. Serum ACE2 activities were plotted as a function of left ventricular ejection fraction (EF), in healthy people (healthy, n = 45, panel A), in patients with preserved EF (hypertensive, n = 239, panel B and in patients with severe reduction in EF (HF - CRT before, n = 65, panel C). Serum ACE2 activities were also plotted as the function of the cardiac morphological parameters end systolic diameter (ESD, panel D) and end diastolic diameter (EDD, panel E) in heart failure patients before CRT (HF – CRT before). Each dot represents values of individual patients. Points were fitted by linear regression. The value P indicates the level of significance of the positive correlation between the plotted parameters, while r represents a measure of the goodness of the fit.

**Figure 4:**
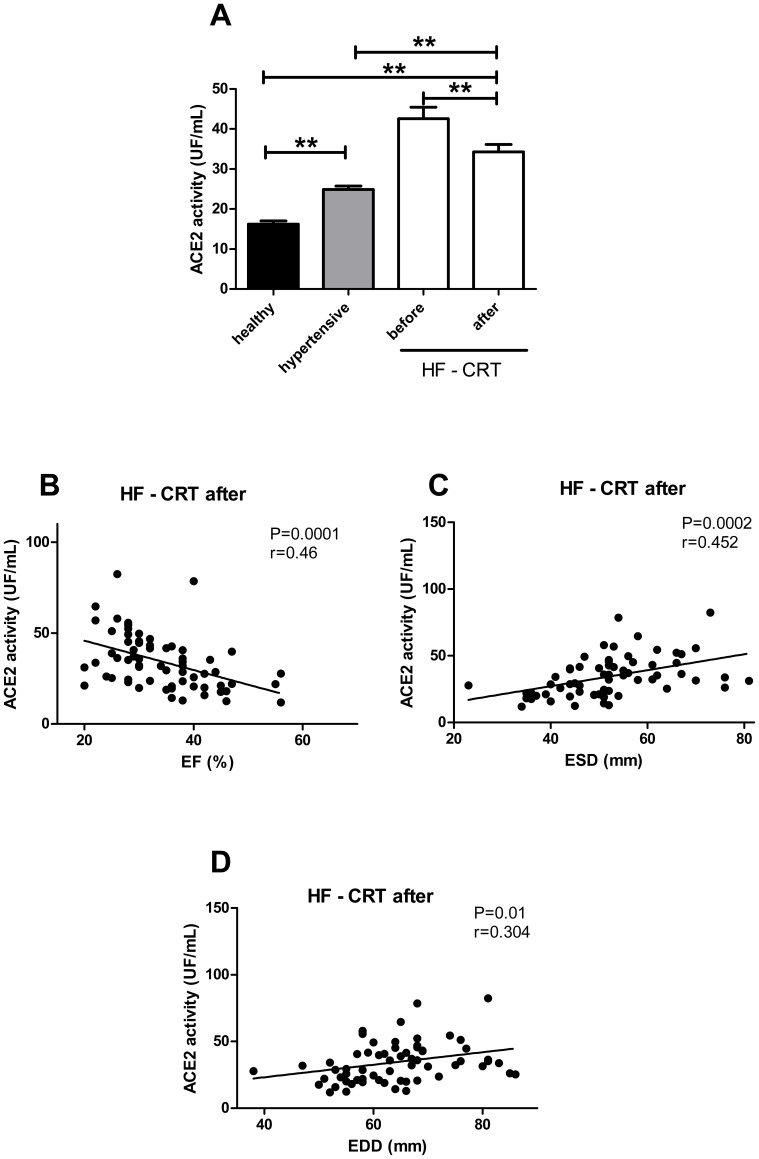
Serum ACE2 activity correlates with reverse remodeling. Serum ACE2 activity was determined in healthy people (healthy, n = 45, filled bar), patients with preserved ejection fraction (hypertensive, n = 239, grey bar) and in patients with severe heart failure before (HF – CRT before) and after (HF – CRT after) cardiac resynchronization therapy (CRT, n = 65, open bars, panel A). Statistical analyses were performed by one-way analysis of variance (ANOVA) followed by Newman-Keuls test for multiple comparisons between groups. Bars represent mean ± S.E.M. Significant differences between values are labeled by asterisks (** P<0.001). In addition, individual values for the patients after CRT (HF – CRT after) are also shown (panels B–D). Serum ACE2 activities were plotted as a function of ejection fraction (EF, panel B), end systolic diameter (ESD, panel C) and end diastolic diameter (EDD, panel D). Plots were fitted by linear regression and the goodness of fits is indicated by the ‘r’ values, while the correlations are represented by the ‘P’ values.

**Figure 5:**
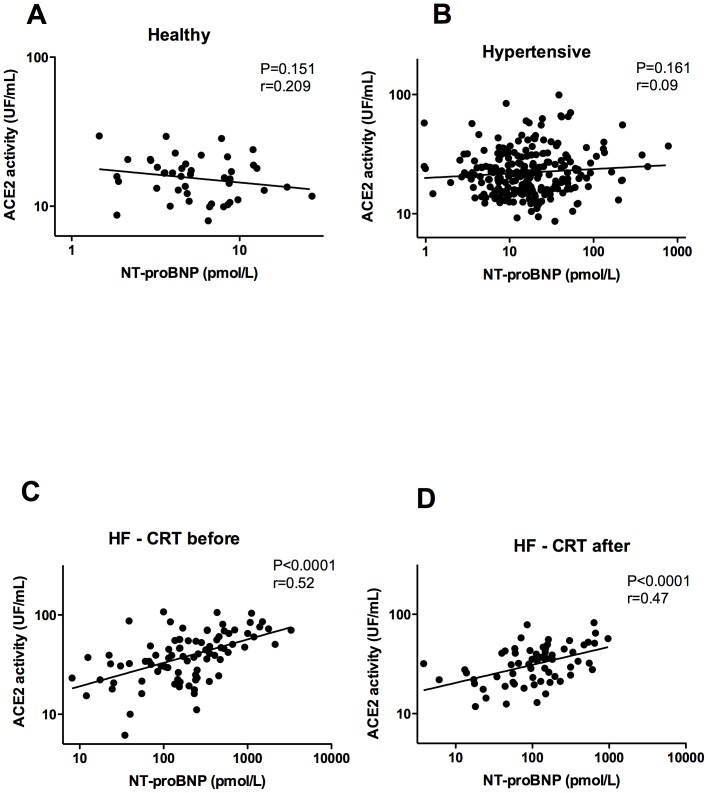
Positive correlation between sACE2 activity and NT-proBNP concentration. Serum ACE2 activities were plotted as the function of NT Pro-Brain Natriuretic Peptide (NT-proBNP) concentrations in various study groups (healthy, n = 45, panel A, hypertensive, n = 239, panel B, HF - CRT before, n = 100, panel C, HF - CRT after, n = 65, panel D). Each dot represents values of individual patients in these plots. Points were fitted by linear regression. The value P indicates the level of significance of the positive correlation between the plotted parameters, while r represents a measure of the goodness of the fit.

**Figure 6:**
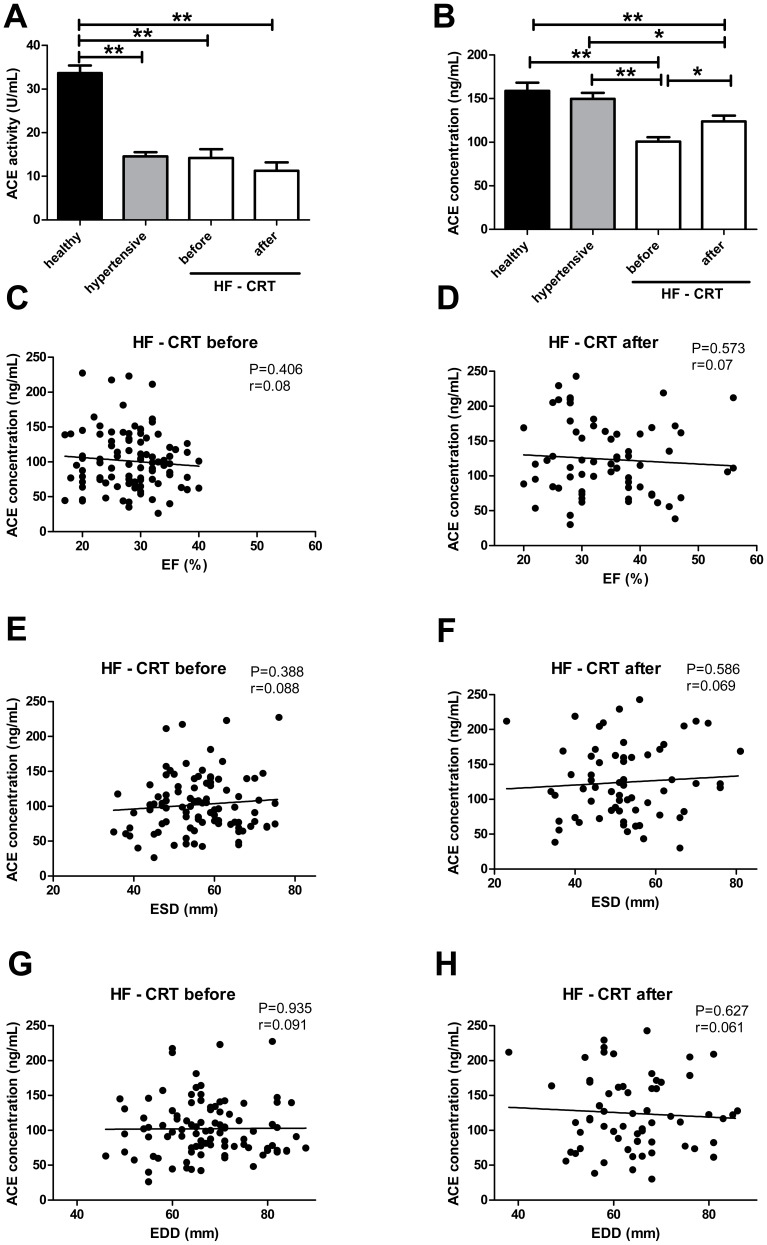
Serum ACE activity and concentration in patients with normal systolic left ventricular function and in patients with systolic heart failure. Serum ACE activity (panel A) and ACE concentration (panel B) were measured in healthy people (n = 45, filled bar), in patients with preserved ejection fraction (hypertensive, n = 40, grey bar) and in patients with severe heart failure before (HF - CRT before) and after cardiac resynchronization therapy (HF - CRT after, n = 65, open bars). Statistical analyses of biochemical measurements was performed by one-way analysis of variance (ANOVA) followed by Newman-Keuls test for multiple comparisons between groups. Bars represent mean ± S.E.M. Significant differences are labeled by asterisks (* P<0.01 and ** P<0.001). Values for the individual patients were also plotted. Serum ACE concentration is showed as a function of the ejection fraction (EF, panels C and D), end systolic diameter (ESD, panels E and F) and end diastolic diameter (EDD, panels G and H). Plots were fitted by linear regression. Values for the goodness of the fits (r) and for the level of correlations (P) are also indicated.

Linear regression analysis was performed to compare sACE2 activity with echocardiographic parameters and NT-proBNP levels (Figures 3–5). Linear regression analysis was performed to compare serum ACE concentration with echocardiographic parameters (Figure 6). P<0.05 was considered to be statistically significant for all the comparisons. All statistical analyses were performed using a commercially available software GraphPad Prism, version 4.0 (GraphPad Software, Inc., San Diego, CA, USA).

## Results

### Baseline characteristics of study groups

Clinical characteristics of the study groups are shown in Table 1. The healthy (systolic/diastolic blood pressure: 122.5±10.9/79.5±9.8 mmHg) and the hypertensive group (optimal medical therapy, systolic/diastolic blood pressure: 134.0±19.9/80.3±11.5 mmHg) consisted of individuals with no apparent symptoms and clinical signs of systolic or diastolic heart failure (Table 1). The CRT group consisted of patients with mild (NYHA II) to severe heart failure (NYHA III–IV) and matched ECG and echocardiographic criteria for biventricular pacemaker implantation. The mean EF in the CRT group was seriously reduced (Table 1) with enlarged end-systolic (ESD, Table 1) and end-diastolic (EDD, Table 1) diameters. In all cases CRT-D device was implanted. Significantly elevated NT-proBNP levels were found in the hypertensive and CRT groups compared to the healthy group. Occurrence of cardiovascular risk factors and medication in the study population is also summarized in Table 1. The pharmaceutical therapy remained unchanged during the study.

### Effects of biventricular pacing on echocardiographic parameters

Hypertensive and heart failure (HF) patients had significantly enlarged initial left ventricular dimensions (ESD, EDD) before cardiac resynchronization therapy (HF - CRT before) compared to healthy individuals (Table 1, Figure 1A and 1B). Severe reduction of left ventricular pumping function was observed as represented by reduced EF in the CRT group (Table 1, Figure 1C). A significant decrease of ESD and EDD (ESD to 51.9±1.4 mm; P<0.001, EDD to 63.9±1.2 mm; P<0.01, Figure 1A and 1B) was found following 6 to 9 months of biventricular pacing (HF – CRT after). Pumping function also improved to a great extent in this group (EF to 34.2±1.1%; P<0.001; Figure 1C). The dP/dt parameter changed in a significant manner as well following CRT (from 498.6±27.1 to 695.4±54.0 mmHg/s; P<0.001; Figure 1D).

### sACE2 activity positively correlates with the clinical status of systolic heart failure patients

A remarkable elevation of sACE2 activity was present in CRT patients before pacemaker implantation (HF – CRT before) compared to healthy people and to the hypertensive patients with preserved left ventricular EF (healthy: 16.2±0.8 UF/mL, hypertensive: 24.8±0.8 UF/mL and HF - CRT before: 30.2±1.7 UF/mL, Figure 2A). Serum ACE2 activity strongly correlated with the clinical condition of patients with severe heart failure (HF - CRT before: NYHAII: 32.3±3.1 UF/mL, NYHAIII: 45.2±2.9 UF/mL, NYHAIV: 64.2±6.2 UF/mL, Figure 2B).

The positive correlation between sACE2 and NYHA classes remained unchanged after pacemaker implantation (HF - CRT after: NYHAI: 25.8±2.5 UF/mL, NYHAII: 35.4±2.3 UF/mL, NYHAIII: 45.9±6.0 UF/mL, Figure 2C), while the clinical status improved in most of the HF-patients.

Angiotensin converting enzyme (ACE) expression was generally lower in heart failure patients before CRT (healthy: 159.0±9.3 ng/mL, hypertensive: 149.6±6.9 ng/mL; HF - CRT before: NYHA II: 102.0±6.4 ng/mL, NYHAIII: 103.7±6.3 ng/mL, NYHAIV: 90.9±10.2 ng/mL, Figure 2D), but this general relationship disappeared after CRT (HF – CRT after: NYHAI: 114.6±10.4 ng/mL; NYHAII: 128.3±8.5 ng/mL; NYHA III: 123.9±22.9 ng/mL, Figure 2E).

### sACE2 activity correlates with hypertension and with the deterioration of left ventricular systolic function

Serum ACE2 activity correlated with the clinical status of HF. Accordingly, we performed a detailed study to identify serum ACE2 as a biomarker of human heart failure. Serum ACE2 activities were plotted as the function of systolic ejection fraction in all study groups (Figure 3A, 3B and 3C). Serum ACE2 activities negatively correlated with EF in hypertensive patients (Figure 3A) similarly to HF patients (Figure 3C). In contrast, no correlation was present between EF and sACE2 activity in healthy individuals (Figure 3B). Moreover, a clear positive correlation was found between serum ACE2 activities and ESD (Figure 3D) and EDD (Figure 3E) in HF patients.

### sACE2 activity correlates with the improvement in left ventricular function

Serum ACE2 activity was higher in hypertensive patients compared to healthy individuals (hypertensive: 24.8±0.9 UF/mL, healthy: 16.2±0.8 UF/mL; P<0.0001, Figure 4A) and further increased in HF patients (HF – CRT before: 42.5±2.9 UF/mL; P<0.0001, Figure 4A), suggesting that sACE2 activity may be a biomarker of cardiovascular disease or imminent heart failure. In this respect, a good biomarker also correlates with the clinical improvement of the disease. Correlation of sACE2 was therefore also investigated upon the clinical improvement after biventricular pacemaker implantation (HF- CRT after). A robust reduction in serum ACE2 activity was found in parallel to the improving cardiac performance and function called reverse remodeling (HF - CRT after: to 34.3±1.9 UF/mL; P<0.001, Figure 4A).

Regression analyses of serum ACE2 as a function of EF after CRT revealed that the correlation remained negative (P<0.01, r = 0.46, Figure 4B), and positive correlation of sACE2 activity to ESD and EDD was also preserved (ESD: P<0.01, r = 0.45; EDD: P = 0.01, r = 0.3, Figure 4C and 4D).

### Correlation of sACE2 activity with NT-proBNP

There was no correlation between sACE2 activity and NT-proBNP in individuals with normal left ventricular systolic function (healthy and hypertensive, Figure 5A and 5B). Serum ACE2 activities positively correlated to NT-proBNP levels in HF patients before CRT (HF – CRT before: P<0.01, r = 0.52, Figure 5C). This positive correlation of sACE2 activity and NT-proBNP was maintained after CRT (HF – CRT after: P<0.01, r = 0.47, Figure 5D).

### Correlation of serum ACE activity and concentration to left ventricular systolic function

ACE activity of the cardiovascular patients (hypertensive: 14.5±0.9 U/mL, HF - CRT before: 14.2±2.0 U/mL and HF - CRT after: 11.3±1.9 U/mL, Figure 6A) were significantly lower than that in healthy individuals (healthy: 33.6±1.7 U/mL, Figure 6A), most probably representing successful ACE inhibitory therapy. Patients with severe systolic heart failure had significantly lower enzyme concentration than patients with preserved EF (healthy: 159.0±9.3 ng/mL, hypertensive: 149.6±6.9 ng/mL and HF - CRT before: 100.5±5.3 ng/mL; P<0.001, Figure 6B). Moreover, serum ACE concentration increased after pacemaker implantation (HF – CRT after: to 123.1±6.5 ng/mL; P<0.01, Figure 6B). Nevertheless, regression analyses of ACE concentration as a function of EF before CRT (HF - CRT before) or after CRT (HF-CRT after) did not show any significant correlations (Figure 6C–6H).

## Discussion

One of the most frequent cardiovascular diseases is hypertension. It leads to secondary cardiomyopathy, coronary artery disease, stroke, peripheral artery disease, chronic nephropathy, neuropathy and many other severe pathologies and ultimately increases mortality. Interestingly, most of the cases (up to 95%) do not have identifiable cause (essential hypertension). It is important to note, that there are effective treatments for hypertension, making it a relatively easily manageable disease, but there is no cure for it, which is probably related to the issue, that the very cause of the disease is not known. As a result, patients are treated and their blood pressure is kept at an acceptable level (target blood pressure is reached by medication), but their background pathology causing the hypertension may be unaffected.

Here we found for the first time that serum ACE2 activities are increased in hypertensive patients (without apparent heart failure, ejection fraction >50%) compared to healthy individuals. Nonetheless, these hypertensive patients already had a barely decreased ejection fraction compared to the healthy population and also featured slightly elevated NT-proBNP levels, suggesting that this population is at imminent heart failure. At this stage of the disease serum ACE2 activity was already elevated although it did not correlate with NT-proBNP levels. It appears therefore, that serum ACE2 activity is another biomarker of imminent heart failure in hypertension. Importantly, sACE2 may also be related to the cause of the disease. It can regulate (decrease) local angiotensin II levels, affecting vascular diameter (vascular smooth muscle located angiotensin receptors) and sympathetic tone (neuronal angiotensin receptors). It may also contribute to the synthesis of angiotensin peptides which may activate the Mas receptor, believed to be beneficial in hypertension. This hypothesis is supported by recent findings suggesting that serum ACE is a rate-limiting step in RAAS: endogenous inhibitors [31], such as serum albumin [32] effectively (>90%) inhibit circulating ACE activity in human.

Although this is the first time when increased sACE2 activity was shown in hypertension in human, the potential connection between sACE2 and regulation of blood pressure is not a new concept. Previous animal data have shown that transgenic sACE2 overexpression in the vessels of SHRSP rats normalizes (reduces) high blood pressure [11]. Modulation of sACE2 activity in the nervous system (overexpression) also attenuated hypertension [12] indicating a possibly protective role of sACE2 in hypertension. Genetic inactivation of sACE2 was again found to be a negative regulator of the RAS in blood pressure control [11] [13] [14]. Moreover, genetic association studies have shown a strong association of sACE2 polymorphisms to hypertension in different human populations [5] [16] [17], although without reference to the actual effect of these polymorphisms on the circulating ACE2 activity. In this respect our study identified sACE2 activity as a biomarker in imminent human heart failure.

The sACE2 was also implicated as a biomarker of definitive heart failure in animal models where absence of sACE2 caused severely impaired cardiac function [19] [20] [15] [30].

There is a wealth of information in human heart failure. Myocardial sACE2 gene expression increased in patients with left ventricular dysfunction including thinning of the left ventricle and severe reduction in cardiac contractility [20]. Burrell et al. observed an increase in sACE2 expression in failing human hearts independently of AT1R blockade [31]. Loss of sACE2 worsened the pathological remodeling and the systolic dysfunction [21] Moreover, sACE2 gene expression increased in tissue samples of patients with left ventricular dysfunction and serum ACE2 activity was elevated and correlated with disease severity [26].

Here we made an effort to directly address the relationship between serum ACE2 activity and cardiac function. Changes in serum ACE2 activity were measured in severe heart failure when cardiac resynchronization therapy (CRT) is indicated [24] [23]. We found significant relationships among plasma ACE2 activity and both left ventricular ejection fraction (EF) and NT-proBNP (Figure 5A) which were reported earlier by Epelman et al. [8]. Our new data therefore repeat these findings. However, we also made a detailed analysis of the relationship between serum ACE2 activity and the echocardiographic systolic parameter EF at the individual patient's level (Figure 3). Serum ACE2 activity negatively correlated with EF in patients with systolic HF (Figure 3C) as well as in hypertensive patients with preserved EF (Figure 3A), but not in healthy volunteers (Figure 3B).

In HF people serum ACE2 activity also correlated with the NT-proBNP concentration, which is the most widely used molecular marker of systolic HF with a reasonably high predictive and prognostic value. It has been suggested that soluble ACE2 levels are independent predictors of a composite end-point consisting of all-cause mortality, cardiac transplantation and heart failure hospitalization [26]. For the first time we have shown that NT-proBNP concentration positively correlates with serum ACE2 activity at the individual patient's level (Figure 5A). This particular observation showed that there is no clearly identifiable patient population with high NT-proBNP and relatively low sACE2 levels, which has been suggested to be a better prognostic factor [26]. Furthermore this kind of relationship still exists when cardiac function improves (Figure 5B).

Elevated circulating ACE2 level in systolic HF raise the question whether serum ACE2 concentration is elevated due to a general increase in sACE2 expression or the increasing soluble ACE2 activity represent a re-distribution of sACE2 from the tissues to the serum. Higher rate of release from the cells may lead to lower tissue sACE2 levels in parallel with increasing serum ACE2 activities in this latter case.

**Figure 7:**
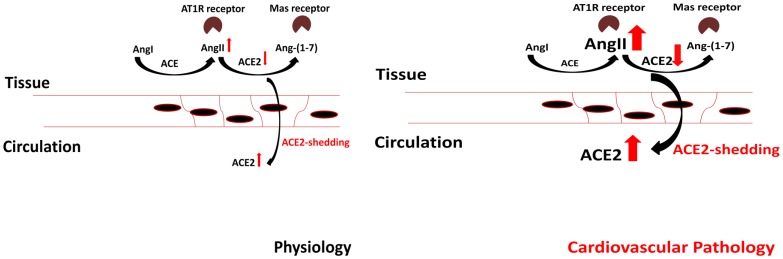
Hypothetic model for RAS regulation in heart failure. Angiotensin I (AngI) is being converted to angiotensin II (AngII) by angiotensin converting enzyme (ACE). AngII is further metabolized to angiotensin 1–7 (Ang-(1–7)) by sACE2. AngII binds to and activate the type 1 AngII receptors (AT1R), while Ang-(1–7) binds to and activate the Mas receptor. Levels of tissue located and soluble forms of these enzymes determine local levels of angiotensin peptides. We hypothesize that a significant redistribution of tissue and soluble ACE and ACE2 occurs in human heart failure which changes are reversible upon improvement of the cardiac function. In particular, sACE2 limits AngII levels under healthy conditions, resulting in a low level of ATR1 stimulation and high Mas receptor stimulation, a condition which is beneficial in cardiovascular physiology. However, ACE2 is probably re-distributed in imminent or definitive heart failure. As a result, its activity increases in the circulating blood, but decreases in the tissues. This result in higher local Ang II levels, higher AT1R stimulation and lower Mas receptor activation. These latter features being characteristic to heart failure.

Data from animal models suggest that sACE2 shedding is stimulated by the tumor necrosis factor-α convertase ADAM17. Moreover, TACE/ADAM17 is upregulated in HF [9]. Therefore, cleavage of membrane sACE2 may be due to the up-regulation of a pathological protease related to proinflammatory mechanisms during myocardial injury, resulting in the decrease of local membrane sACE2 levels and an increase in tissue AngII levels, while decreasing Ang-(1–7). These changes in the local concentrations of AngII and Ang-(1–7) can significantly contribute to remodeling and to the deterioration of cardiac function (Figure 7).

A considerable part of currently used biomarkers are related to a specific disease indirectly. Here we tested the soluble ACE2 activity for the first time when cardiac function is being improved, but no change in drug therapy occurs. We found that soluble ACE2 activity correlated well with the improvement of cardiac function after implantation of a biventricular pacing device (CRT). In particular, improving EF after CRT correlated negatively with serum ACE2 activities (Figure 4A and 4B), improvement of cardiac morphological parameters correlated positively with sACE2 activities (ESD, Figure 4C and EDD, Figure 4D).

An effort was also made to correlate the soluble ACE levels to cardiovascular disease. ACE expression changed with the improvement of cardiac function. In particular, soluble ACE concentration was lower before pacemaker implantation than in the healthy individuals and in patients with hypertension with preserved ejection fraction and it increased in parallel with the improvement of cardiac function (Figure 6B). In spite of increased soluble ACE concentrations soluble ACE activity did not change in the diseased populations (Figure 6A) suggesting effective therapeutical inhibition of ACE, besides to the recently shown stabilizing effect of serum albumin on ACE activity [30]. Regression analyses of ACE concentration with EF, ESD and EDD before and after CRT groups did not show any correlation in spite to the promising correlation between soluble ACE concentration and cardiovascular disease (Figure 6C–6H). Moreover, although ACE expression was lower in patients with heart failure before CRT, this general relationship disappeared after CRT, when an improvement was observed in the clinical parameters. These data suggested that ACE expression is not related ultimately to the clinical status and severity of heart failure.

There is a significant interest about HF biomarkers. Many candidates were proposed, including peptides, enzymes, receptors of inflammatory system, changes in the extracellular matrix of myocytes, oxidative stress and neurohormones [27], [32]. Clinical biomarker testing related to HF is aimed to identify possible underlying causes, to confirm the presence or absence of HF and to estimate the severity and the risk of disease progression, according to the consensus document of The National Academy of Clinical Biochemistry of the US [33]_ENREF_30. An ideal biomarker does not only indicate the pathology but also takes elemental part of that. Our data suggest that sACE2 is involved in disease pathomechanism, it correlates well with disease severity and even with regression of the disease so it is a candidate for HF biomarker.

Based on these data we hypothesize that redistribution of ACE and in particular sACE2 contribute to the pathomechanism of HF. We found that soluble ACE concentration decreases, while soluble ACE2 concentration increases in systolic HF suggesting an opposite change in the tissue located forms of these enzymes. These changes promote AngII formation in the tissues, contributing to AngII related pathologies. Therapeutical interventions leading to improvement of cardiac function reverse the changes in soluble enzyme concentrations (Figure 7). It is not clear yet, whether these changes are consequences of HF, or important contributors to the progression and reversion of the disease. Nonetheless, this latter is supported by the clinical effectiveness of ACE inhibitor drugs in the management of HF. Inhibition of the redistribution of ACE enzymes therefore represents a new therapeutic strategy to counterbalance tissue RAS activation.

Having said that we have to point out that this hypothesis is limited by the lack of the direct measurement of tissue ACE and sACE2 expression. It may also be possible that changes in the serum concentrations of ACE and sACE2 represent changes in the expression level of these proteins, rather than their redistribution. This issue needs to be clarified by further clinical studies.

## Supporting Information

Figure S1 **Specificity of Mca-APK(Dnp) hydrolysis as a measure of sACE2 activity.** Mca-APK(Dnp) was incubated with a single test human serum sample for 120 min in the presence of 0–3 μM sACE2 inhibitor DX600, as detailed in the methods. DX600 completely inhibited Mca-APK(Dnp) hydrolysis in a concentration dependent manner in accordance with its inhibitory activity on sACE2.
(TIFF)

## References

[1] Nicholls MG, Richards AM, Agarwal M (1998) The importance of the renin-angiotensin system in cardiovascular disease. J Hum Hypertens 12: 295–299. 9655650 10.1038/sj.jhh.1000638

[2] Ferrario CM (1990) The renin-angiotensin system: importance in physiology and pathology. J Cardiovasc Pharmacol 15 Suppl 3S1–5. 1691411

[3] Donoghue M, Hsieh F, Baronas E, Godbout K, Gosselin M, et al (2000) A novel angiotensin-converting enzyme-related carboxypeptidase (ACE2) converts angiotensin I to angiotensin 1–9. Circ Res 87: E1–9. 10969042 10.1161/01.res.87.5.e1

[4] Santos RA, Simoes e Silva AC, Maric C, Silva DM, Machado RP, et al (2003) Angiotensin-(1–7) is an endogenous ligand for the G protein-coupled receptor Mas. Proc Natl Acad Sci U S A 100: 8258–8263. 12829792 10.1073/pnas.1432869100 PMC166216

[5] Zhong J, Basu R, Guo D, Chow FL, Byrns S, et al.. (2010) Angiotensin-converting enzyme 2 suppresses pathological hypertrophy, myocardial fibrosis, and cardiac dysfunction. Circulation 122: 717–728, 718 p following 728. 10.1161/CIRCULATIONAHA.110.955369 20679547

[6] Kostenis E, Milligan G, Christopoulos A, Sanchez-Ferrer CF, Heringer-Walther S, et al (2005) G-protein-coupled receptor Mas is a physiological antagonist of the angiotensin II type 1 receptor. Circulation 111: 1806–1813. 15809376 10.1161/01.CIR.0000160867.23556.7D

[7] Gallagher PE, Ferrario CM, Tallant EA (2008) Regulation of ACE2 in cardiac myocytes and fibroblasts. Am J Physiol Heart Circ Physiol 295: H2373–2379. 18849338 10.1152/ajpheart.00426.2008 PMC2614534

[8] Epelman S, Shrestha K, Troughton RW, Francis GS, Sen S, et al (2009) Soluble angiotensin-converting enzyme 2 in human heart failure: relation with myocardial function and clinical outcomes. J Card Fail 15: 565–571. 19700132 10.1016/j.cardfail.2009.01.014 PMC3179261

[9] Lambert DW, Yarski M, Warner FJ, Thornhill P, Parkin ET, et al (2005) Tumor necrosis factor-alpha convertase (ADAM17) mediates regulated ectodomain shedding of the severe-acute respiratory syndrome-coronavirus (SARS-CoV) receptor, angiotensin-converting enzyme-2 (ACE2). J Biol Chem 280: 30113–30119. 15983030 10.1074/jbc.M505111200 PMC8062222

[10] Wang W, Bodiga S, Das SK, Lo J, Patel V, et al (2012) Role of ACE2 in diastolic and systolic heart failure. Heart Fail Rev 17: 683–691. 21638102 10.1007/s10741-011-9259-x

[11] Rentzsch B, Todiras M, Iliescu R, Popova E, Campos LA, et al (2008) Transgenic angiotensin-converting enzyme 2 overexpression in vessels of SHRSP rats reduces blood pressure and improves endothelial function. Hypertension 52: 967–973. 18809792 10.1161/HYPERTENSIONAHA.108.114322

[12] Yamazato M, Yamazato Y, Sun C, Diez-Freire C, Raizada MK (2007) Overexpression of angiotensin-converting enzyme 2 in the rostral ventrolateral medulla causes long-term decrease in blood pressure in the spontaneously hypertensive rats. Hypertension 49: 926–931. 17325232 10.1161/01.HYP.0000259942.38108.20

[13] Gurley SB, Allred A, Le TH, Griffiths R, Mao L, et al (2006) Altered blood pressure responses and normal cardiac phenotype in ACE2-null mice. J Clin Invest 116: 2218–2225. 16878172 10.1172/JCI16980 PMC1518789

[14] Wysocki J, Ye M, Rodriguez E, Gonzalez-Pacheco FR, Barrios C, et al (2010) Targeting the degradation of angiotensin II with recombinant angiotensin-converting enzyme 2: prevention of angiotensin II-dependent hypertension. Hypertension 55: 90–98. 19948988 10.1161/HYPERTENSIONAHA.109.138420 PMC2827767

[15] Zhong J, Yan Z, Liu D, Ni Y, Zhao Z, et al (2006) Association of angiotensin-converting enzyme 2 gene A/G polymorphism and elevated blood pressure in Chinese patients with metabolic syndrome. J Lab Clin Med 147: 91–95. 16459167 10.1016/j.lab.2005.10.001 PMC7127450

[16] Lu N, Yang Y, Wang Y, Liu Y, Fu G, et al (2012) ACE2 gene polymorphism and essential hypertension: an updated meta-analysis involving 11,051 subjects. Mol Biol Rep 39: 6581–6589. 22297693 10.1007/s11033-012-1487-1

[17] Patel SK, Wai B, Ord M, MacIsaac RJ, Grant S, et al (2012) Association of ACE2 genetic variants with blood pressure, left ventricular mass, and cardiac function in Caucasians with type 2 diabetes. Am J Hypertens 25: 216–222. 21993363 10.1038/ajh.2011.188

[18] Der Sarkissian S, Huentelman MJ, Stewart J, Katovich MJ, Raizada MK (2006) ACE2: A novel therapeutic target for cardiovascular diseases. Progress in Biophysics & Molecular Biology 91: 163–198. 16009403 10.1016/j.pbiomolbio.2005.05.011

[19] Bodiga S, Zhong JC, Wang W, Basu R, Lo J, et al (2011) Enhanced susceptibility to biomechanical stress in ACE2 null mice is prevented by loss of the p47(phox) NADPH oxidase subunit. Cardiovasc Res 91: 151–161. 21285291 10.1093/cvr/cvr036 PMC3151662

[20] Goulter AB, Goddard MJ, Allen JC, Clark KL (2004) ACE2 gene expression is up-regulated in the human failing heart. BMC Med 2: 19. 15151696 10.1186/1741-7015-2-19 PMC425604

[21] Yamamoto K, Ohishi M, Katsuya T, Ito N, Ikushima M, et al (2006) Deletion of angiotensin-converting enzyme 2 accelerates pressure overload-induced cardiac dysfunction by increasing local angiotensin II. Hypertension 47: 718–726. 16505206 10.1161/01.HYP.0000205833.89478.5b

[22] Lehmann HI, Wolke C, Malenke W, Rohl FW, Hammwohner M, et al.. (2012) Enzymatic activity of DPIV and renin-angiotensin system (RAS) proteases in patients with left ventricular dysfunction and primary prevention implantable cardioverter/defibrillator (ICD). Int J Cardiol. 10.1016/j.ijcard.2012.09.083 23063136

[23] Dickstein K, Cohen-Solal A, Filippatos G, McMurray JJ, Ponikowski P, et al (2008) ESC Guidelines for the diagnosis and treatment of acute and chronic heart failure 2008: the Task Force for the Diagnosis and Treatment of Acute and Chronic Heart Failure 2008 of the European Society of Cardiology. Developed in collaboration with the Heart Failure Association of the ESC (HFA) and endorsed by the European Society of Intensive Care Medicine (ESICM). Eur Heart J 29: 2388–2442. 18799522 10.1093/eurheartj/ehn309

[24] Dickstein K, Vardas PE, Auricchio A, Daubert JC, Linde C, et al (2010) 2010 Focused Update of ESC Guidelines on device therapy in heart failure: an update of the 2008 ESC Guidelines for the diagnosis and treatment of acute and chronic heart failure and the 2007 ESC guidelines for cardiac and resynchronization therapy. Developed with the special contribution of the Heart Failure Association and the European Heart Rhythm Association. Eur Heart J 31: 2677–2687. 20801924 10.1093/eurheartj/ehq337

[25] Vickers C, Hales P, Kaushik V, Dick L, Gavin J, et al (2002) Hydrolysis of biological peptides by human angiotensin-converting enzyme-related carboxypeptidase. J Biol Chem 277: 14838–14843. 11815627 10.1074/jbc.M200581200

[26] Epelman S, Tang WH, Chen SY, Van Lente F, Francis GS, et al (2008) Detection of soluble angiotensin-converting enzyme 2 in heart failure: insights into the endogenous counter-regulatory pathway of the renin-angiotensin-aldosterone system. J Am Coll Cardiol 52: 750–754. 18718423 10.1016/j.jacc.2008.02.088 PMC2856943

[27] Patel SK, Velkoska E, Burrell LM (2013) Emerging markers in cardiovascular disease: where does ACE2 fit in? Clin Exp Pharmacol Physiol. 10.1111/1440-1681.12069 23432153

[28] Ronca-Testoni S (1983) Direct spectrophotometric assay for angiotensin-converting enzyme in serum. Clin Chem 29: 1093–1096. 6303627

[29] Beneteau B, Baudin B, Morgant G, Giboudeau J, Baumann FC (1986) Automated kinetic assay of angiotensin-converting enzyme in serum. Clin Chem 32: 884–886. 3009054

[30] Fagyas M, Úri K, Mányiné IS, Daragó A, Boczán J, et al.. (2014) New perspectives in the renin-angiotensin-aldosterone system (RAAS) III: endogenous inhibition of angiotensin converting enzyme (ACE) provides protection against cardiovascular diseases. PLoS One. 10.1371/journal.pone.0093719 PMC3972147 24690767

[31] Fagyas M, Úri K, Mányiné IS, Daragó A, Boczán J, et al.. (2014) New perspectives in the renin-angiotensin-aldosterone system (RAAS) I: endogenous angiotensin converting enzyme (ACE) inhibition. PLoS One. 10.1371/journal.pone.0087843 PMC3972180 24691160

[32] Fagyas M, Úri K, Mányiné IS, Fülöp GÁ, Csató V, et al.. (2014) New perspectives in the renin-angiotensin-aldosterone system (RAAS) II: albumin suppresses angiotensin converting enzyme (ACE) activity in human. PLoS One. 10.1371/journal.pone.0087844 PMC3972182 24691203

[33] Crackower MA, Sarao R, Oudit GY, Yagil C, Kozieradzki I, et al (2002) Angiotensin-converting enzyme 2 is an essential regulator of heart function. Nature 417: 822–828. 12075344 10.1038/nature00786

[34] Burrell LM, Risvanis J, Kubota E, Dean RG, MacDonald PS, et al (2005) Myocardial infarction increases ACE2 expression in rat and humans. Eur Heart J 26: 369–375 discussion 322–364. 15671045 10.1093/eurheartj/ehi114

[35] van Kimmenade RR, Januzzi JL Jr (2012) Emerging biomarkers in heart failure. Clin Chem 58: 127–138. 22086968 10.1373/clinchem.2011.165720

[36] Tang WH, Francis GS (2007) Cardiac resynchronization therapy in New York Heart Association class IV heart failure: it is all about selection. Circulation 115: 161–162. 17228011 10.1161/CIRCULATIONAHA.106.669879

