# Supplementary figures and images for: New Perspectives in the Renin-Angiotensin-Aldosterone System (RAAS) IV: Circulating ACE2 as a Biomarker of Systolic Dysfunction in Human Hypertension and Heart Failure
Source: PLoS One. 2014 Apr 1;9(4):e87845. doi: 10.1371/journal.pone.0087845 (PMC3972189; doi:10.1371/journal.pone.0087845)

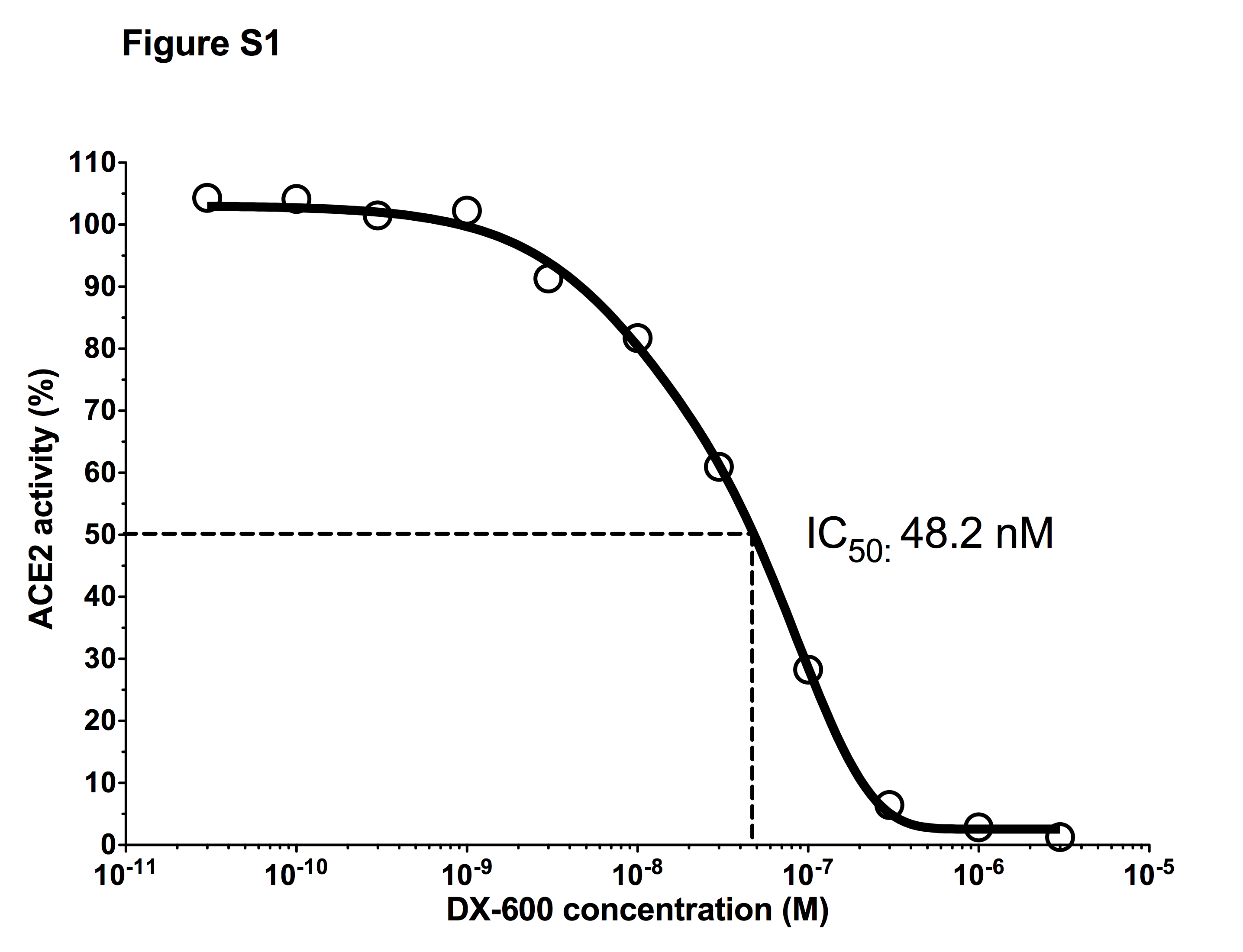

Supplement: Figure S1 — Specificity of Mca-APK(Dnp) hydrolysis as a measure of sACE2 activity. Mca-APK(Dnp) was incubated with a single test human serum sample for 120 min in the presence of 0–3 μM sACE2 inhibitor DX600, as detailed in the methods. DX600 completely inhibited Mca-APK(Dnp) hydrolysis in a concentration dependent manner in accordance with its inhibitory activity on sACE2. (TIFF) [file pone.0087845.s001.tiff]
